# Census of economic evaluations in primary prevention 2014–2019: a scoping review protocol

**DOI:** 10.1186/s13643-020-01315-8

**Published:** 2020-03-24

**Authors:** Hannah Jackson, Alan Shiell

**Affiliations:** School of Psychology and Public Health, La Trobe University, Bundoora, Victoria 3086 Australia

**Keywords:** Scoping review, Protocol, Census, Economic evaluation, Public health practice, Primary prevention, Preventive health services, Preventive medicine

## Abstract

**Background:**

A large proportion of the burden of disease is preventable, yet investment in health promotion and disease prevention programmes remains a small share of the total health budget in many countries. The perception that there is paucity of evidence on the cost-effectiveness of public health programmes is seen as a barrier to policy change. The aim of this scoping review is to conduct a census of economic evaluations in primary prevention in order to identify and map the existing evidence.

**Methods:**

This review is an update of a prior census and will include full economic evaluations of primary prevention programmes conducted in a community-based setting that were published between 2014 and 2019. The search of electronic databases (MEDLINE and Embase, and NHS-EED for 2014) will be supplemented by a search for grey literature in OpenGrey and a search of the reference lists of reviews of economic evaluations identified in our searches. Retrieved citations will be imported into Covidence® and independently screened in a two-stage process by two reviewers (abstracts and full papers). Any disagreements on the eligibility of a citation will be resolved by discussion with a third reviewer. Included studies will then be categorised by one independent reviewer according to a four-part typology covering the type of health promotion intervention, the risk factor being tackled, the setting in which the intervention took place and the population most affected by the intervention. New to this version of the census, we will also document whether or not the intervention sets out specifically to address inequalities in health.

**Discussion:**

This review will produce an annotated bibliography of all economic evaluations plus a report summarising the current scope and content of the economic evidence (highlighting where it is plentiful and where it is lacking) and describing any changes in the type of economic evidence available for the various categories of disease prevention programmes since the last census. This will allow us to identify where future evaluative efforts should be focused to enhance the economic evidence base regarding primary prevention interventions.

**Systematic review registration:**

Registration is being sought concurrently.

## Background

The case for investing in public health and disease prevention continues to gain momentum, with researchers and policy-makers increasingly arguing for a reallocation of resources from treatment of acute disease to public health interventions aimed at the prevention of disease [1–5]. Despite the evidence that much of the burden of disease is preventable [5–7], spending on prevention remains a small proportion of the total health budget in many OECD countries [8]. This phenomenon is exacerbated by the perception that economic evidence to support investment in disease prevention interventions is lacking. This is true in some instances, such as the cost-effectiveness of preventive interventions in low- and middle-income countries [9], but there is a substantial body of evidence summarising the cost-effectiveness of numerous public health interventions, including seminal work in the WHO’s ‘Best Buys’ document [10], Public Health England’s work on the return on investment and cost-effectiveness of public health programmes [11] and the Assessing Cost-Effectiveness in Prevention (ACE-Prevention) study [12].

An appreciation of the existing economic evidence—its amount, its scope and its content (essentially documenting where the economic evidence is plentiful and where it is lacking)—would be helpful to support both priority setting in public health and the setting of future research agendas in the economics of public health [9, 13].

Our first census of economic evaluations of primary prevention interventions in population health examined literature published between 1990 and 2001 [14]. We found that 90% of the economic evidence examined interventions that addressed the biological or behavioural determinants of health. In contrast, there was hardly any evidence that evaluated population health advocacy or interventions that addressed the social and economic determinants of health. An update covering the historic period 2002 to 2013 is in progress.

This review protocol describes the methods we will use to update the census of economic evaluations in health promotion and disease prevention by examining studies published in the period 2014 to 2019, and to categorise the identified publications in a manner similar to that used in the previous work. This will allow us to report on the growth in the economics evidence base and any changes in its scope and coverage.

## Methods

### Eligibility

Studies will be included in the census if they report a full economic evaluation (i.e. cost-effectiveness, cost-consequence, cost-utility or cost-benefit analysis) as defined by Drummond et al. [15] of at least one primary prevention intervention. All types of study will be considered, including economic evaluations based on data from randomised controlled trials, observational studies, data obtained from large administrative databases and economic modelling studies. Articles must be published in English between the years 2014 and 2019 inclusive, and report an economic evaluation of primary prevention that was conducted in a community setting. This will include interventions that take place in hospital outpatient clinics and primary care interventions as well as community-based and population-wide interventions.

Studies with the following characteristics will be excluded from the review:

- Pilot studies
- Citations with no associated abstract
- Papers that are only available in abstract form (e.g. conference proceedings)
- Systematic reviews of economic evaluations
- Secondary and tertiary prevention interventions
- Studies conducted in a hospital inpatient setting

We have made two changes to the inclusion and exclusion criteria used in the first census [14].

First, we have relaxed the requirement that the intervention being evaluated had to be pertinent to practice in a developed country. This had proved difficult to operationalise consistently other than in an overly simplistic way based on the country of origin. Second, we will exclude studies conducted in in-patient hospital settings where the risks being guarded against are a consequence of the patient’s admission to hospital or the treatment they receive there. This change will help retain the focus on primary prevention.

### Search strategy

We will use a slightly modified version of the search strategy employed in the original census to take into account subsequent improvements in search methodologies. The search strategy for the original census was developed de novo by the authors. In this instance, we have taken on board insights provided by both Glanville et al., for the Canadian Agency for Drugs and Technologies in Health [16], and the work of Sassi and colleagues [17]. These sources show the results from various search filter trials, to examine both sensitivity and specificity in order to determine an efficient strategy.

Resource constraints mean that we are limited in the number of databases that we can search and we have opted to go with both MEDLINE and Embase. The overlap between these two databases varies according to the research topic, although commentators agree that a comprehensive search should incorporate both databases [18, 19]. Details of the search strategies we will use for both databases are provided in the Appendix. We will also search NHS-EED for 2014 (the only year in our time frame for which this database was active). The electronic search strategy will be supplemented by a search for grey literature using ‘OpenGrey’ and by hand searching the reference lists of review articles.

### Data management and screening

The retrieved citations will be imported into Covidence®. At the first stage, citations (title and abstract) will be screened for eligibility in two steps, first to identify any economic evaluations, and second to determine whether the evaluation includes a primary prevention intervention conducted in a community setting. If there is any doubt about the eligibility of a citation, it will be moved forward to the next stage of screening. Full-text articles will then be retrieved for all citations that have progressed to stage two of the screening process. These will be assessed for final eligibility according to the aforementioned eligibility criteria. Two reviewers will independently screen each citation. Where there is a discrepancy between the two reviewers, a third reviewer will be consulted and a final decision will be made after discussion.

### Data extraction

Data extraction and initial categorisation of the studies will be completed by one independent reviewer. A sample of studies will be subject to second blinded review to examine reliability. Any uncertainty as to how to classify a particular intervention will be resolved through discussion between reviewers. Data will be extracted from the included articles using a predefined template. As in the original census, citations will be classified according to a four-part typology, covering:

1. The type of health promotion activity/area of strategic focus
2. The risk factor that the intervention is addressing
3. The setting of the primary prevention intervention
4. The population most affected by the intervention

In this round, we will also add a fifth classification to document whether the intervention specifically sets out to reduce health inequalities, and if so how it does this. Finally, we will document the type of economic evaluation (e.g. cost-effectiveness or cost-utility) and the quadrant(s) of the cost-effectiveness acceptability curve that the results fall into [20].

For part one (type of health promotion activity), studies will be classified according to the five key areas of health promotion practice defined by the World Health Organization’s Ottawa Charter for Health Promotion [21]. In addition to these five key areas, we have added a sixth category ‘clinical–preventive’ to cover clinical interventions that have a health-promoting objective, as was done in the original census publication. Consequently, the six categories describing the health promotion activity in the economic evaluation are as follows:

1. Building healthy public policy
2. Creating supportive environments
3. Strengthening community actions
4. Developing personal skills
5. Reorienting health services
6. Clinical–preventive

For part two (the risk factor that the intervention addresses), studies will be classified according to the World Health Organization’s definition of risk factors as the ‘social, economic or biological status, behaviours or environments which are associated with or cause increased susceptibility to a specific disease, ill health, or injury’ [22]. The five categories we will use to describe the risk factors addressed by the health promotion activity are therefore:

1. Biological
2. Behaviour
3. Environment
4. Social
5. Economic

For part three (the setting of the primary prevention intervention), we will base our categories on the WHO’s definition of settings as ‘the place or social context in which people engage in daily activities in which environmental, organizational and personal factors interact to affect health and wellbeing’ [22].

For part four (population targeted by the intervention), studies will be classified into one of three groups according to the approach used to identify the population of interest:

1. Life stage (e.g. infant, pregnant women)
2. Social (e.g. gay men, obese, travellers)
3. Whole population (e.g. all residents in a specified geographic location)

New to this version of the census is classification of studies according to whether or not the intervention being evaluated specifically addresses inequalities in health. Based on the typology promulgated by Whitehead [23], studies will be classified according to the strategy being adopted (strengthening individuals, strengthening communities, improving living and working conditions, promoting healthy macro-policies or none). We will also note whether or not the intervention targets one or more marginalised groups and whether or not the authors report the results of the evaluation in terms of any change in inequality [24].

### Synthesis of data

Once all of the citations have been classified according to the five-part typology, we will count the number of studies in each category, cross-tabulate the results to look for patterns among the classifications and report on areas in which the evidence is plentiful, areas that may require a greater research focus in the future, and compare the results to the findings of the last census. We will report the number of economic evaluation studies of primary prevention interventions that were published in each year and compare our results to the numbers and the trajectory that was shown in the first census to quantify changes in the evidence base.

As in the previous census, the quality of the economic evidence is not a consideration for inclusion or exclusion and so this will not be assessed here.

## Discussion

### Limitations and challenges of the review

Our aim is to document all published economic evaluations (a census), to identify and map the existing evidence and to act as a repository for policymakers and practitioners seeking evidence on the cost-effectiveness of prevention that might subsequently be subject to critical appraisal. In the previous census, the small number of studies made it feasible to do this and classify all studies according to the criteria described above. The field is expanding, however, and with exercises like ACE-Prevention, a single exercise resulting in 150 economic evaluations [12], we anticipate a much larger number of economic evaluations. Thus, it may not be possible to classify all studies this time. Once the searches are completed and the number of eligible studies is determined, we will consider whether we can categorise all studies or whether we will need to examine a random sample.

Our aim is to collect information on all published economic evaluations in primary prevention/health promotion, using the definition promulgated by the World Health Organization, which regards primary prevention as being action directed towards preventing the initial occurrence of a disorder [25], including actions that modify the social and economic determinants of health. We encountered difficulties maintaining this definition in the original census, in large part because it is difficult to determine with any consistency when a risk factor ends and the first clinical signs of disease begins [26]. If anything, this problem has increased in subsequent years with the increasing prevalence of chronic conditions, and we anticipate further challenges navigating this definitional grey zone. Remembering that the one of the purposes of the census is to act as a repository of evidence for potential use by public health decision-makers, the principle that will guide our decisions is whether the intervention being evaluated would conceivably sit within the purview of a public health agency or department within government.

## Conclusion

This review will update understanding of where the economic literature on primary prevention strategies is plentiful (and thus a possible focus for critical appraisal and systematic review) and where it is lacking, where future evaluative efforts could be focused to enhance the evidence base. Such an overview will provide policy-makers and public health practitioners with relatively easy access to the economic evidence, which might then, after critical appraisal of its quality, be used to inform the allocation of resources.

## Data Availability

Not applicable.

## Appendix

MEDLINE search strategy

1. exp “quality adjusted life year”/

2. qaly*.mp.

3. economic evaluation.mp.

4. costs.tw.

5. (cost effectiv* or cost utilit* or cost benefit*).mp.

6. 1 or 2 or 3 or 4 or 5

7. pc.fs.

8. exp “vaccines”/

9. exp “vaccination”/

10. exp health promotion/

11. exp smoking cessation/

12. exp preventive medicine/

13. exp accident prevention/

14. exp “public health practice”/

15. exp “Tobacco Use Cessation”/

16. exp Health Education/

17. exp Nutrition Therapy/

18. prevent*.mp.

19. 7 or 8 or 9 or 10 or 11 or 12 or 13 or 14 or 15 or 16 or 17 or 18

20. 6 and 19

21. (animals not (humans and animals)).sh.

22. 20 not 21

23. limit 22 to (english language and yr="2014 - 2019")

Embase search strategy

1. exp “quality adjusted life year”/

2. exp “economic evaluation”/

3. qaly*.mp.

4. costs.tw.

5. (cost effectiv* or cost utilit* or cost benefit*).mp.

6. 1 or 2 or 3 or 4 or 5

7. pc.fs.

8. exp vaccine/

9. exp “vaccination”/

10. exp “smoking cessation”/

11. exp “health program”/

12. exp “public health service”/

13. exp “community program”/

14. exp diet therapy/

15. exp health education/

16. prevent*.mp.

17. 7 or 8 or 9 or 10 or 11 or 12 or 13 or 14 or 15 or 16

18. 6 and 17

19. limit 18 to (english language and yr="2014 - 2019")

## Abbreviations

ACE: Assessing cost-effectiveness
WHO: World Health Organization
